# Improvements in executive functions by domain-specific cognitive training in youth elite soccer players

**DOI:** 10.1186/s40359-024-02017-9

**Published:** 2024-10-02

**Authors:** Florian Heilmann, Simon Knöbel, Franziska Lautenbach

**Affiliations:** 1 Movement Science Lab, Institute for Sport Science, Martin-Luther University Halle-Wittenberg, von-Seckendorff-Platz 2, Halle (Saale), 06120 Germany; 2 Institute for Sport Psychology and Sport Pedagogy, Leipzig, Germany; 3 Department of Sport Psychology, Institute for Sport Science, Humboldt-University of Berlin, Berlin, Germany

**Keywords:** Executive functions, Cognitive training, Soccer, Youth athletes

## Abstract

This study examined the impact of sport-specific cognitive training (CT) on executive functions (EFs) in youth soccer players. Thirty-one athletes (13–15 years) participated, 13 in the intervention group (IG) and 18 in the control group (CG). The IG underwent an 8-week soccer-focused CT program, while the CG maintained regular training. The assessments included working memory, inhibition, and cognitive flexibility tasks. The results revealed no significant improvements in EFs in the IG compared to those in the CG. Both groups showed enhanced cognitive flexibility, possibly due to general cognitive development or learning effects. The study suggested that an 8-week sport-specific CT may not enhance EFs in young soccer players, potentially due to a ceiling effect in highly skilled athletes. These findings should be considered when designing cognitive training programs for athletes, and future research could explore the optimal duration of such programs.

## Introduction

In team sports, various skills and abilities, such as endurance, power, speed, and flexibility, as well as tactical and technical skills, are essential for superior performance [1, 2]. In recent years, cognitive functions and abilities have increasingly become a research focus [3, 4]. Primarily, executive functions (EFs; inhibition, working memory, cognitive flexibility) are related to sports performance [5–9].

Executive functions are higher-level cognitive processes critical for goal-directed behavior, particularly in dynamic environments [10]. There is a lack of knowledge about whether the general capacity of these functions can be enhanced through targeted cognitive training (CT). This paper presents a comprehensive study on the impact of domain-specific CT on the EFs of youth soccer athletes. Leveraging a rigorous experimental design, we enrolled a cohort of young soccer players and systematically exposed them to a series of cognitive training methods tailored to on-field decision-making and cognitively challenging scenarios. The interventions focused on enhancing working memory, inhibitory control, and cognitive flexibility. Pre- and postintervention assessments were conducted using a battery of validated EF tasks. The findings could help practitioners decide whether they should implement CT in their practice.

### Executive functions

Core EFs are often characterized by Diamond [10]: inhibition, working memory, and cognitive flexibility. *Inhibition* refers to the ability of an individual to suppress or stop a motor response that has already been initiated or to prevent an automatic response from occurring [11]. The cognitive mechanism that is in charge of momentarily storing and modifying information in the mind is known as *working memory*. It is a crucial component of short-term memory [12] and plays a critical role in many cognitive processes, including problem-solving, decision-making, and learning [12]. *Cognitive flexibility* refers to shifting one’s thinking or attention between different concepts, tasks, or mental sets in response to changing environmental demands. It involves adapting behavior to new situations, updating beliefs or strategies when necessary, and considering alternative perspectives or solutions [13].

### Approaches to EFs in sports

The *expert-performance approach* studies the athlete under sport-specific conditions (i.e., domain-specific or sport-specific) and tries to explain peak performance in sports. The second common approach to investigating the relevance of cognition in sports is the *cognitive skills component approach* [14, 15]. This approach focuses on athletes’ higher cognitive functions (EFs and various subdomains of visual attention). Recent studies within this approach explore fundamental visual and cognitive functions or skills in sport-unspecific contexts (i.e., domain-generic).

EFs are decisive in team sports such as soccer because they involve higher-order cognitive processes that enable individuals to plan, organize, initiate, and regulate their actions to achieve a specific goal [14]. For example, in soccer, players must constantly decide when to pass the ball, inhibit irrelevant information, sustain attention to relevant cues (*inhibition*), decide when to shoot, and where to move on the field. They must also pay attention to the movements of their teammates and opponents, know where they are (*working memory and updating*), switch between different tactics, and adjust their strategies based on the changing circumstances of the game (*cognitive flexibility*; [16, 17].

The unity-diversity framework [18], as an essential concept in understanding the relation and differentiation between the constructs of EFs, posits that EFs are unified in the sense that they are all related to the control and regulation of behavior. However, they also exhibit diversity in that they can be distinguished based on their unique neural substrates [19], developmental trajectories, and behavioral correlates. In the context of sports performance, the unity-diversity framework suggests that different EFs may be more critical for success in certain sports than in others, depending on the specific demands of the activity [20]. Sports that necessitate prompt decision-making and swift adaptation to evolving circumstances, for instance, might place greater emphasis on cognitive flexibility. In contrast, those who demand sustained attention and memory for complex strategies may require working memory (i.e., sailing).

Recently, a debate about the ecological validity of EF tasks started. Ball et al. [21] postulated that brief laboratory tasks may be inadequate for describing the depth and breadth of EFs in sports, especially in team sports. Several working groups are reviewing the first approaches to developing sport-specific EF tasks [22–24].

### Cognitive training in athletes

“The content of cognitive training is the active design of both thought and imagination processes so that performance-enhancing cognitions can be retrieved according to the situation” [25]. The definition thus also includes cognitions that are developed in such a way that there is a high probability that they can have a performance-enhancing effect in game situations within sports games. In the literature, a distinction is made between a domain-generic or domain-unspecific CT and a domain-specific or sport-specific CT. This implies transferring cognitive skills to even a “far” or “near” context. The generalization of skills or functions learned or trained in different areas is known as a transfer of skills. Transferring skills between related domains is called “near” transfer.

On the other hand, “far” transfer occurs weakly or unrelatedly between domains. It is widely known in the psychological literature that although distant transfer is significantly more intriguing to research or accomplish, it is much less common than near transfer. Nevertheless, there are studies on cognitive transfer in sports.

### Domain generic CT and “far” transfer

If cognitive training has a domain-generic character, there must be a “far transfer” to the particular sport [15]. A far transfer means that the specific skill or function trained in a domain generic CT could be transferred to a particular context or a sports situation. In this case, working memory training, such as playing memory, would impact athletic performance while climbing. Moreover, sports practice could improve cognitive skills or functions measured in a laboratory task [20]. This would mean that training soccer players, for example, would affect performance in an EF task.

In general, there is a debate about the effectiveness of CT, especially concerning commercial products [26]. There are inconsistent findings about the efficacy of cognitive training and brain training apps (i.e., Cognifit, Cogmed, Lumosity, Neurotracker; for a review, see, e.g [27, 28]. , . In addition, the effects could differ between different age groups. The authors of the relevant studies explain the differences in effects with the domain specificity of the games or applications. Often, the effects are not translated into the real-world context.

Furthermore, studies focusing on athletes with a domain-generic approach to cognitive training have shown inconsistent effects on performance in EF tasks [15].

Harris et al. [29] reported limited support for far-transfer effects from CT to sporting tasks. They explain these restricted effects by the target of the studies because they did not target the sporting environment. Scharfen & Memmert [30] reported positive effects of 3D multiple object tracking training (i.e., NeuroTracker) on trained ability (i.e., multiple object tracking; near transfer) but not on perceptual-cognitive functions such as visual clarity or inhibition.

Romeas et al.’s [31] study investigated the transfer of training effects of a domain-generic cognitive intervention on sports performance. This study demonstrated the beneficial effects of using the NeuroTracker, a cognitive training tool, on soccer players’ self-reported decision-making and passing accuracy.

Generally, the far transfer of cognitive skills is seen critically [32]. There are inconsistent findings on whether sporting activities foster the development of EFs. Beavan et al. [33, 34] found no support for the assertion that EFs are closely associated with, for example, football experience in a series of investigations carried out inside an organization that uses cognitive assessment and training in high-level footballers. Moreover, meta-analyses have shown inconsistent results [20, 35].

### Domain-specific CT and “near” transfer

Thus, the transfer of cognitive training measures is mainly a near transfer and not a far transfer, which means that domain-generic cognitive training fails to improve cognitive performance in a sporting context. In fact, Beavan et al. [33, 34] demonstrated that a participant’s age likely plays a significant role in mediating the association between football and competence.

For example, Scharfen and Memmert [15] examined the effects of 3-D multiple object tracking training (Neurotracker) on executive functions and attention. The results demonstrated small gains in a few other tasks (i.e., inhibition, visual clarity) but significant near-transfer benefits to the training skill (i.e., MOT). The study of Moen et al. (2018) could not confirm several effects on EFs, but the findings are in line with the study of Scharfen and Memmert [15] regarding the training effects of the Neurotracker software itself. In the study of Heilmann et al. [26], professional youth soccer athletes played a smartphone game to facilitate EFs. There were no significant effects on performance in the EF tasks.

As seen in the studies, several ceiling effects may affect the improvements achieved by cognitive interventions for athletes [36]. Ludyga et al. [37] postulated that young and middle-aged adults have not been the focus of previous studies, and ceiling effects were anticipated. This is because interventions were expected to be less efficient due to limited reserves for adaptations.

Nevertheless, evidence shows that EFs are essential in team sports [38, 39]. As a result, there is a fair amount of interest in assessing athletes’ training potential for these functions, especially in soccer [30].

However, there is an inconsistent and small amount of research on the impact of *domain-generic* and *domain-specific cognitive training* in athletes [30].

### The present study

A recent study examined the effects of a short-term (i.e., eight weeks) domain-specific cognitive training program on EFs (i.e., inhibition, working memory, and cognitive flexibility) in youth soccer players. The findings could provide information on the possibility of training EFs in high-performance youth athletes, especially those with demands in open skills (i.e., soccer). In addition, it could fill the knowledge gap about whether EFs could be improved or if they are only determined by the age-related development of prefrontal structures of the brain [34].

These findings could be important for researchers and practitioners to evaluate the opportunities to decide whether developing sport-specific EF training programs is beneficial [40].

We hypothesized that the domain-specific cognitive training intervention would improve EF performance (i.e., inhibitory control, working memory, cognitive flexibility) in the intervention group in comparison to the control group.

## Methods

### Participants

An a-priori statistical power analysis (t-test, two dependent means) was conducted for sample size estimation based on the effect size of [41]; *N* = 11, *r* = .71). Thus, with an alpha of 0.05 and a power of 0.95 for two groups, the sample size needed for the current study (calculated with G*Power 3.1) was *N* = 23. We tried to recruit 30 to 35 players for the study to compensate for a possible drop out. Thirty-one soccer players of a youth soccer academy (male; the highest league in the respective age group) aged 13 to 15 years (*M* = 14.30; *SD* = 0.59) participated voluntarily in the study. For organizational reasons, players from sports boarding schools were assigned to the intervention group (*n* = 13). The active control group (*n* = 18) had an individual technical skills training session of the same duration as the control group. Because we did not want to exclude anyone from the intervention or the control group, the sample was fully measured and the data processed.

**Fig. 1:**
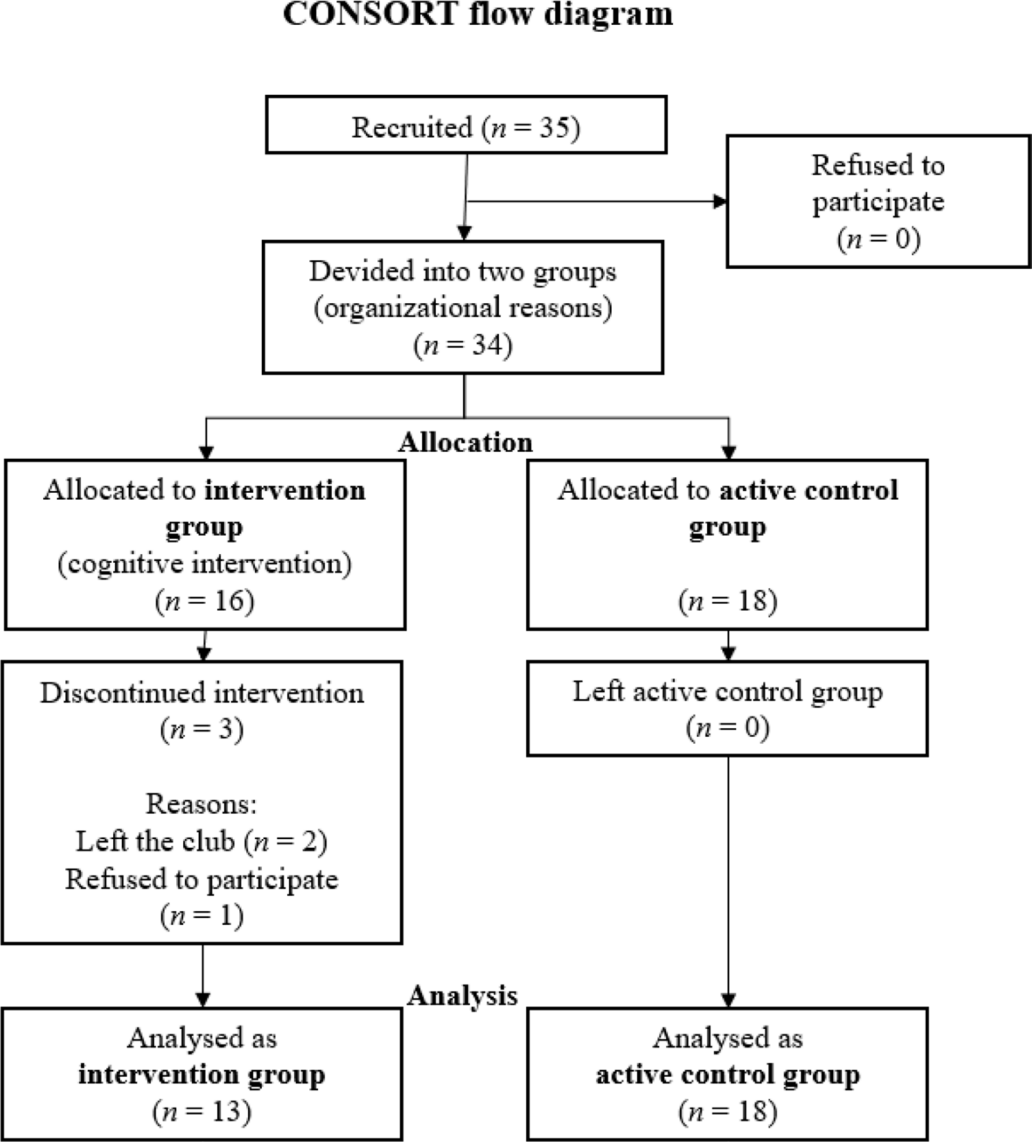
CONSORT flow diagram of the randomization,* allocation*, and analysis of participants

Three players were excluded because they did not complete the intervention, as they either left the club (*n* = 2) or decided not to participate in the study (*n* = 1; see Fig. 1).

The Declaration of Helsinki and the APA’s ethical guidelines were followed in the study protocol. The study was approved by the university’s ethical committee (approval number of the ethical committee of Humboldt University of Berlin: HU-KSBF-EK_2022_0018). Informed consent was obtained from every participant as well as from their legal representatives.

### Measurements

Using Inquisit Lab 6 (Millisecond Software LLC, Seattle, WA, USA) on a 17-inch screen and a QWERTZ keyboard, computerized tasks were used to measure the EFs [9].

### Inhibitory control

**Fig. 2:**
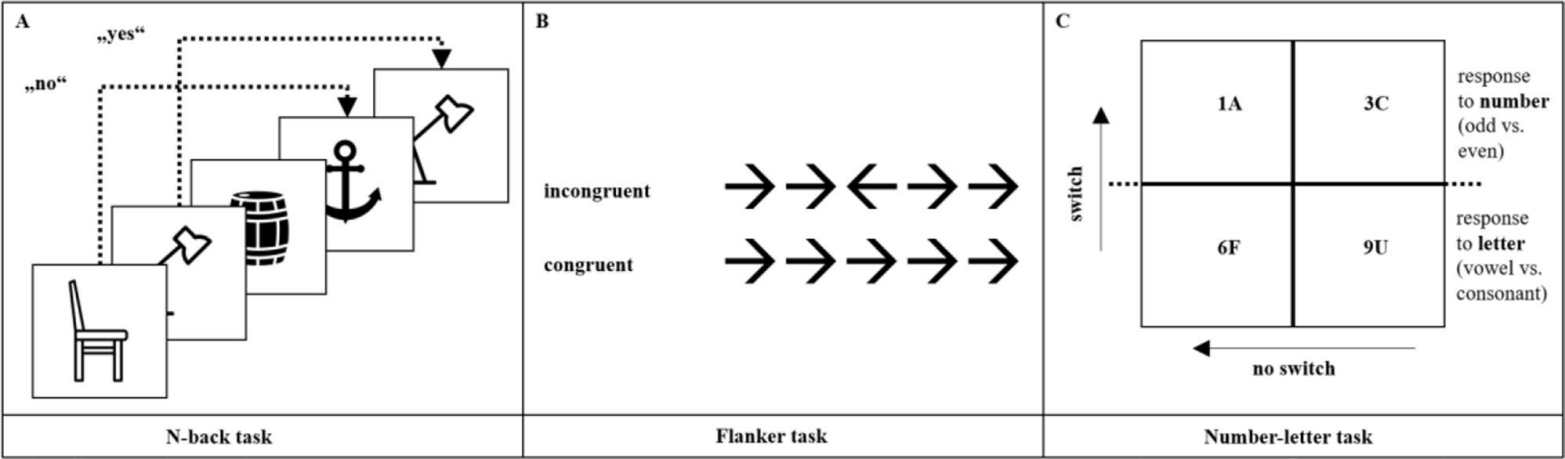
Stimuli presentation of tasks to assess EFs: (**A**) 3-back task, (**B**) Flanker task with incongruent and congruent trials, and (**C**) number-letter task with switch and no-switch trials

For the Flanker task [42, 43], subjects had to respond to a stimulus consisting of five black arrows on a white background. The middle of the five arrows was the one that the subjects had to focus on. The task was to press the “I” button when the arrow was pointing to the right and the “E” button when the arrow was pointing to the left. There were congruent trials in which all the arrows pointed in one direction, incongruent trials in which the middle arrow pointed in one direction, and trials in which all the other arrows pointed in the opposite direction (distraction arrows; please see Fig. 2). The Flanker task included four training trials followed by 72 test trials (24 incongruent and 48 congruent trials). We measured the response times for correct trials and the accuracy of the responses as the outcome variables for congruent and incongruent trials of the Flanker task. Moreover, the influence of differences in stimulus incongruency on response times between congruent and incongruent stimuli was measured using the Flanker effect. Low values for the Flanker effect reflect good inhibitory control of participants. The task has shown high reliability coefficients in previous research [44]; congruent trials [response time]: *r* = .856; incongruent trials [response time]: *r* = .879).

### Working memory

A computerized version of the n-back task, the 3-back task [45]; Fig. 2), was used to examine participants’ working memory. For the test, participants were presented with emotionally neutral images and asked to indicate whether the image they were currently viewing had been presented to them three images previously. They had to press the “A”-button if this was the case. Participants completed 23 test attempts, followed by 46 target attempts. The response times for the correct attempts and the accuracy of the answers were measured as outcome variables. A high number of correct answers and a short response time indicate good working memory. Kirchner et al. [46] showed good task reliability (response time for correct trials: *r* = .950).

### Cognitive flexibility

To assess the subjects’ cognitive flexibility, the number-letter task (derived from the alternating runs switch task [47] was modified as a computer-based test. A 2 × 2 matrix with a number-letter pair shown in one of the four matrix cells was presented to the participants (see Fig. 2). The numbers or letters moved clockwise through the four boxes of the matrix. In the top two boxes, the subjects had to respond to the letter and indicate whether it was a vowel (pressing the “I” key) or a consonant (pressing the “E” key). For this condition, the participants had 24 practice trials and 32 regular trials. For the bottom two boxes, participants had to respond to the digits by pressing the “I” key for even stimuli and the “E” key for odd stimuli as quickly as possible (24 practice stimuli, 32 regular stimuli). Participants were given 24 practice trials in which only the letter or number was presented and 24 in which both letters and numbers were given. Finally, 64 target trials were conducted, including 32 switch trials (switching from focusing on numbers to letters or vice versa) and 32 nonswitch trials (further focusing on numbers or letters). The number-letter task response times and response accuracy for stimuli with and without task switching were computed. The so-called switch costs and the variations in response times between the switch and no-switch experiments were used to measure the participants’ cognitive flexibility. Short response times, lower switching costs, and high accuracy indicate greater cognitive flexibility. The task demonstrated strong reliability in earlier studies. [48]; switch cost [response time]: *r* = .676; recurrence trials [response time]: *r* = .773).

### Intervention

A domain-specific cognitive training intervention was developed for the study. The exercises were adapted from Memmert [49] and Nowak & Vestberg [17] and were implemented in an 8-week training plan with two training units per week and a duration of 40–50 min (2 × 20–25 min per training unit). The training units consisted of exercises that train EFs in a soccer-specific environment. The supplemental material (Sup. 1) provides a further description of the training intervention via the following link: https://osf.io/aucdf/?view_only=2ec940fb40554f6da7c3545c348a3336.

### Procedures

All participants or their legal representatives provided informed consent. The participants underwent pre- and posttests (EF tests) at their training facilities (the quiet room of the functional building of the youth academy). The participants had to complete the tasks on a 17-inch screen and a QWERTZ keyboard. The EF tasks were conducted in a randomized order. The tests were administered in October 2022 (pretest, the first week of the intervention period) and November 2022 (posttest, the eighth week). Informed consent was given to the participants after they were briefed on the procedure. They then completed the cognitive tasks, which took approximately 30 min. To minimize the effects of physical exertion, the players were assessed between 10:00 a.m. and 4:00 p.m., one hour prior to training. After the pretest, the experimenter explained the intervention to the participants. The participants also had to complete a questionnaire.

### Data preparation

A preliminary filter for the flanker task for inhibition eliminated all trials with erroneous replies (pre: 2.64%; post: 3.23%). To accommodate extreme outcomes, a second filter was removed (pre and post: 0%) in all trials with response times lower than 200 ms or more than 1.750 ms (e.g [23]. , . A final filter (pre: 1.34%; post: 1.38%) eliminated response times that were +- 3 SD outside the individual mean. For the Flanker task, one player was excluded because of an incomplete dataset.

For the *3-back task*, a filter was used to identify all missed target trials (pre: 36.43%; post: 41.14%). In addition, all responses to nontarget trials (i.e., false alarms) were determined (pre: 17.88%; post: 15.71%), and the corresponding response times were excluded. Thus, the mean response time is calculated only for target trials where participants answered correctly. Furthermore, a second filter was applied to exclude all response times that deviated ± 3 SD from the individual mean (pre and post: 0.0%). The percentage of successful responses to target trials and the frequency of false alarms were calculated to estimate the overall accuracy in the first step. The rate of successful target trials minus the proportion of unsuccessful nontarget trials (i.e., false alarms) was then calculated. We also analyzed how many target trials were missed (see [22]). For the 3-back task, two players were excluded because of incomplete datasets.

A first filter for the *number-letter task* that was designed to gauge cognitive flexibility eliminated all trials with wrong answers (pre: 15.82%; post: 9.17%). To adjust for severe results, the second filter (pre: 7.43%; post: 3.65%) and third filter (pre: 0.42%; post: 1.11%) were employed. No datasets were excluded from the analysis of the number-letter task.

### Statistical analysis

Analysis of variance (ANOVA-MR) was used even though our data were not normally distributed because the procedure was resistant to violated normality test assumptions (e.g [50]). , . There were no outliers with an interquartile range greater than 1.5 to exclude. We used a modified Bartlett (MB) test to check for heteroscedasticity as an application requirement for the ANOVA. The distribution met the criteria.

To assess the effects of the intervention on inhibition, we conducted a 2 (time: pre vs. post) x 2 (group: IG [intervention group] vs. CG [control group]) multivariate analysis of variance (MANOVA) that included response time parameters from the flanker task (i.e., response time for incongruent trial) to determine whether inhibition (Flanker task) changed over time as a result of the intervention. We conducted separate 2 (time: pre vs. post) x 2 (group: IG vs. CG) ANOVAs for accuracy measures for the Flanker task because they were not linked with response time characteristics.

Since accuracy and response time were not significantly correlated with one another for the 3-back task, two separate 2 (time: pre vs. post) x 2 (group: IG vs. CG) ANOVAs were computed for the effect of the intervention on working memory.

We adapted the procedure for the parameters describing cognitive flexibility. To investigate the effect of the intervention on cognitive flexibility, we used a 2 (time: pre vs. post) x 2 (group: IG vs. CG) MANOVA using accuracy and response time parameters as the dependent variables.

We then used the Bonferroni correction procedure to conduct a post hoc analysis (univariate ANOVA and t-tests). SPSS 28 (SPSS, Chicago, Illinois, United States) was utilized for the statistical analysis. The significance criterion was set at *p* < .05.

## Results

Table 1 presents all the descriptive information. There were no differences in baseline measurements for all EF tasks.

**Table 1:** Results of executive function measurements

Executive function	Task	Parameter	Intervention (mean ± SD)		Control (mean ± SD)		ANOVA (sig. results)
			Pre	Post	Pre	Post	
Inhibition	Flanker task	congruent [ms]	441.83 (82.89)	433.00 (92.79)	413.05 (69.53)	411.25 (63.93)	
		incongruent [ms]	470.82 (101.91)	464.77 (98.47)	440.05 (83.17)	439.33 (71.71)	
		Flanker effect [ms]	28.99 (24.64)	31.77 (24.96)	26.99 (25.30)	28.09 (17.46)	
		total [%]	96.99 (2.11)	96.79 (2.27)	97.61 (2.61)	96.76 (2.62)	
		congruent [%]	98.78 (1.58)	98.56 (1.71)	98.50 (1.94)	97.92 (2.50)	
		incongruent [%]	93.40 (6.00)	93.24 (5.31)	95.83 (5.20)	94.42 (5.00)	
Working Memory	3-back task	total [ms]	710.32 (275.23)	733.45 (245.74)	817.89 (300.03)	854.70 (340.08)	
		total [%]	38.21 (14.42)	38.23 (13.45)	35.18 (18.38)	43.32 (14.92)	
Cognitive flexibility	Number-letter task	no switch [ms]	1075.50 (214.60)	882.79** (142.30)	1043.53 (233.48)	882.50** (153.39)	time: *F*[29] = 631.14, *p* < .001, *η*_p_^2^ = 0.956 (ω^2^ = 0.488)
		switch [ms]	1416.43 (238.12)	1271.34** (203.28)	1323.61 (254.15)	1202.50** (260.93)	time: *F*[29] = 8.49, *p* = .007, *η*_p_^2^ = 0.226 (ω^2^ = 0.163)
		switch cost [ms]	340.94 (218.69)	388.55 (164.67)	280.08 (199.73)	320.00 (199.30)	
		accuracy (no switch) [%]	92.09 (5.21)	94.60* (4.59)	85.63 (9.35)	92.33* (8.72)	time: *F*[29] = 13.563, *p* < .001, *η*_p_^2^ = 0.319 (ω^2^ = 0.224)
		accuracy (switch) [%]	78.36 (15.32)	88.96 (10.10)	80.65 (11.43)	86.13* (10.34)	time: *F*[29] = 7.818, *p* = .009, *η*_p_^2^ = 0.212
		accuracy (switch cost) [%]	-13.73 (12.39)	-5.65 (7.80)	-4.98 (10.55)	-6.20 (6.02)	

### Inhibition

For the response time parameters of the Flanker task, the results showed no significant main effects for factor group (*F* [3, 31] = 0.56, *p* = .460, *η*_p_^2^ = 0.020), factor time (*F* [3, 31] = 0.53, *p* = .473, *η*_p_^2^ = 0.019) or the interaction (*F* [3, 31] = 0.43, *p* = .516, *η*_p_^2^ = 0.015). We did not observe further significant effects for the accuracy parameters for the factors group (*F* [3, 31] = 0.26, *p* = .616, *η*_p_^2^ = 0.009), time (*F* [3, 31] = 2.449, *p* = .080, *η*_p_^2^ = 0.257) or interaction (*F* [3, 31] = 1.213, *p* = .280, *η*_p_^2^ = 0.042).

### Working memory

There were no significant main effects for factor group (*F* [3, 31] = 0.27, *p* = .607, *η*_p_^2^ = 0.010), factor time (*F* [3, 31] = 0.02, *p* = .872, *η*_p_^2^ = 0.001), or the interaction (*F* [3, 31] = 2.924, *p* = .099, *η*_p_^2^ = 0.101) for the response time parameters in the 3-back task. We did not observe any significant effects for the accuracy parameters for the factors group (*F* [3, 31] = 0.30, *p* = .589, *η*_p_^2^ = 0.011), time (*F* [3, 31] = 1.125, *p* = .299, *η*_p_^2^ = 0.041), or interaction (*F* [3, 31] = 0.00, *p* = .992, *η*_p_^2^ = 0.000).

### Cognitive flexibility

We showed main effects for the response time parameters of the number-letter task for the factor time (*F* [3, 31] = 8.49, *p* = .007, *η*_p_^2^ = 0.226). Furthermore, we did not observe significant effects for the factors group (*F* [3, 31] = 1.88, *p* = .181, *η*_p_^2^ = 0.061) or interaction (*F* [3, 31] = 0.28, *p* = .602, *η*_p_^2^ = 0.009). We confirmed the effects on the response time [ms] for the switch and no-switch trials (switch: *F* [29] = 8.49, *p* = .007, *η*_p_^2^ = 0.226; no-switch: *F* [29] = 631.14, *p* < .001, *η*_p_^2^ = 0.956) but not on the switch costs (*F* [29] = 1.04, *p* = .316, *η*_p_^2^ = 0.035).

Furthermore, there was a significant effect of time on the accuracy parameters (*F* [3, 31] = 9.91, *p* = .004, *η*_p_^2^ = 0.255). Again, there were no significant effects for the factors group (*F* [3, 31] = 0.54, *p* = .470, *η*_p_^2^ = 0.018) or interaction (*F* [3, 31] = 1.027, *p* = .319, *η*_p_^2^ = 0.034) for the accuracy parameters. The effects could be confirmed for the response time [ms] for the switch and no-switch trials (switch: *F* [29] = 7.818, *p* = .009, *η*_p_^2^ = 0.212; no-switch: *F* [29] = 13.563, *p* < .001, *η*_p_^2^ = 0.319) but not for the switch costs (*F* [29] = 0.85, *p* = .363, *η*_p_^2^ = 0.029).

## Discussion

The present study aimed to investigate whether a domain-specific sport-specific cognitive training (CT) intervention (8 weeks, two times per week, 20–30 min) tailored to training aspects of EFs could positively impact or transfer the EF task performance of young soccer players in a laboratory setting. Contrary to our expectations, our results showed no significant effect of the CT intervention on the EFs of the youth athletes. Interestingly, cognitive flexibility performance increased in both groups over time.

Previous studies have shown that there are advantages regarding the EFs of soccer players compared with control groups without a profession in sports [15] or novices in soccer [5, 39]. Fransen [32] postulates that the authors of the mentioned studies erroneously concluded that there is a strong relation between EF performance and future success (*far transfer*), for example, in soccer. Nevertheless, they omitted the factor of calendar age. Beavan et al. [34] and Heilmann et al. [9] showed that the relationship between soccer expertise and EFs is firmly determined by participants’ age.

For example, in a regression model, Heilmann et al. [9] showed that only the combination of calendar age and inhibitory control could explain variance in the soccer performance of participants rated by coaches. Thus, the possibility of developing EFs through cognitive training should be considered critically, and the participant’s age must be considered.

Nevertheless, the potential of domain-specific CTs has not been extensively studied. Romeas et al. [31] examined the effects of multiple object tracking (MOT) CT on the sport-specific performance of soccer players. This study demonstrated possible far-transfer effects, such as the beneficial effects of a MOT CT, on soccer players’ self-reported decision-making and passing accuracy. Our findings do not align with these results because we could not demonstrate evidence of a far transfer of cognitive performance. Furthermore, in a recent study, we examined the effects of a domain-specific CT intervention on performance on EF tasks. The measurement provides more objective information about cognitive functions than a self-report. This unique study factor could lead to a lack of alignment between the findings.

Nevertheless, they can also be examined in the opposite direction (e.g., improvements in EF performance in a laboratory setting by domain-specific CT interventions). The findings of Scharfen & Memmert [30] underpin a *near but not broad transfer* of the effects of CT on EFs. They examined the impact of MOT training on EFs and further visual-cognitive functions.

According to a study by Moen et al. [52], athletes from a variety of sports, including martial arts, handball, soccer, biathlon, alpine skiing, and Paralympic sports, such as sled hockey, badminton, and table tennis, exhibit transfer effects from 3D MOT training (Neurotracker) on executive brain functions, including alerting, orienting, executive control, inhibition, shifting, and updating. These studies have shown transfer effects from domain generic cognitive training to sports performance, and they are not in line with recent findings. Nevertheless, some studies suggest no noticeable effects of domain-generic CT on EF performance in soccer players [26].

The effectiveness of CT on soccer athletes or any athlete, in general, can vary depending on several factors, including the specific nature of the cognitive training program, the athletes’ baseline cognitive functions, and the training goals. From a practical perspective, the causal relationship between the affordances in open-skill sports and EFs is logically understandable. For example, a soccer player lines up a pass when he or she sees a clear, open passing way to a teammate poised to score. However, an opponent’s quick movement immediately blocks the passing path. A skilled player will then stifle the initial passing motion and might opt to pass to a different teammate or take advantage of the opening left by the advancing opponent to get closer to the goal. Therefore, it seems that soccer players need strong inhibitory control and other higher-order cognitive abilities to perform at the best level [32]. Nevertheless, the effects could be marginal or nonexistent because of the lack of transferability.

The following factors could affect the effectiveness of CT for athletes, especially soccer players. A cognitive training program may not have been designed to address the specific cognitive skills required for soccer (*training specificity*). Cognitive skills, including reaction time, decision-making, spatial awareness, and more, are diverse. Athletes may already possess high levels of cognitive function, especially in areas related to their sport. If their cognitive abilities are already well developed, it can be challenging to achieve substantial improvements (*baseline of cognitive functions*). Eight weeks may not be sufficient to observe significant improvements in EFs (*training duration*; [26]. CT often requires a longer and more consistent effort to produce noticeable results. Furthermore, athletes have different learning curves and responses to training [33]. Some athletes may benefit from CT more than others, depending on their characteristics and learning styles (*individuality*). Future studies should capture data on fitting with preferred learning styles (questionnaires). The effectiveness of a cognitive training program can vary widely depending on the quality of the program (*training quality*), the engagement of the athletes, and the expertise of the trainers or coaches. Virtual reality training in the context of CT should be considered for future studies, as this tool can be used for further standardization. An essential factor to consider is that the EF measurement is ecologically valid (*valid cognitive assessment*). In the current study, a domain-generic EF task was chosen to examine the participants’ EF performance. Some improvements in cognitive skills may not be readily apparent in the field and may require more nuanced assessments to detect. As Furley et al. [51] postulated in their critical review, the objective measures of EFs use laboratory-based measures or computer-based methods, and they are relatively remote from everyday life. This is also the case for the measurements of EFs and sporting activities. The measurements could only display cognitive functions with a far transfer to sports performance. Musculus et al. [23] and Knöbel et al. [22] advanced in this direction. They validated a sport-specific (soccer) EF task to examine inhibition, working memory, and cognitive flexibility by using sport-specific stimuli (soccer players turning to the left/right side instead of arrows for the flanker task) and responses (shooting a ball to a left or right goal instead of pressing the left or right button).

### Study limitations

Some limitations of the present study must be acknowledged. First, the sample size is small, and in conjunction with the fact that the sample examined probably does not show a large variance in their sport-specific performance (homogenous group), the performance difference in EF tasks, and the impact of the sport-specific CT intervention. Second, the training quality and particularly the improvement in the cognitive tasks of the CT were not controlled for. In the future, this could help to determine an improvement in training and demonstrate a transfer effect to the EF test. For this purpose, a virtual reality setting could be helpful because the process could be tracked via different scores for performance, and the intervention could be standardized. Third, as mentioned in the discussion section, cognitive measurement plays an important role. The measurement could have an ecologically valid character. In the current study, the EF tasks were based on domain-generic stimuli (arrows, symbols uncharged with emotions, digits, and numbers) and domain-generic response options (pressing a button on a keyboard). One could assume that a measurement using sport-specific stimuli and responses would have shown different results. The ecologically valid tests could have led to a more effortless transfer between the ecologically valid intervention and the tests (near transfer). Furthermore, EFs are never retrieved in isolation during soccer training or competitive games. Players experience psychological (i.e., pressure) and physiological (i.e., running distance) demands and stressors that potentially affect their EFs. Thus, the question remains about how diagnostics under neutral resting conditions relate to sport-specific EFs.

## Conclusions

In total, sports performance is influenced by various factors, including physical conditioning, technical skills, and team dynamics. Cognitive training, while essential, is just one component of overall performance, and its effects may not be isolated from other factors.

To improve the effectiveness of CT for soccer athletes, it is essential to consider the specific cognitive skills needed for soccer, tailor training programs accordingly, and provide adequate time for athletes to adapt and show improvements. Additionally, assessing the effectiveness of such training may require using appropriate metrics and considering individual differences among athletes. Ecologically valid tests for examining EFs in a sport-specific setting could help to understand the “near” and “far transfer” paradigms [52]. If EFs are trained in a sport-specific setting, EF-tests must also be more ecologically valid or sport-specific (i.e., a modified Go/No-Go task). In future research, virtual reality could be essential in developing such measures (training and assessments).

## Data Availability

The data that support the findings of this study are openly available at https://osf.io/aucdf/?view_only=2ec940fb40554f6da7c3545c348a3336.
